# A Support Vector Machine-Based Approach for Bolt Loosening Monitoring in Industrial Customized Vehicles

**DOI:** 10.3390/s23115345

**Published:** 2023-06-05

**Authors:** Simone Carone, Giovanni Pappalettera, Caterina Casavola, Simone De Carolis, Leonardo Soria

**Affiliations:** Department of Mechanics, Mathematics and Management, Polytechnic University of Bari, Via Orabona n. 4, 70125 Bari, Italy; simone.carone@poliba.it (S.C.); caterina.casavola@poliba.it (C.C.); simone.decarolis@poliba.it (S.D.C.); leonardo.soria@poliba.it (L.S.)

**Keywords:** bolt looseness detection, support vector machines, vibration, fault diagnosis, structural health monitoring

## Abstract

Machine learning techniques have progressively emerged as important and reliable tools that, when combined with machine condition monitoring, can diagnose faults with even superior performance than other condition-based monitoring approaches. Furthermore, statistical or model-based approaches are often not applicable in industrial environments with a high degree of customization of equipment and machines. Structures such as bolted joints are a key part of the industry; therefore, monitoring their health is critical to maintaining structural integrity. Despite this, there has been little research on the detection of bolt loosening in rotating joints. In this study, vibration-based detection of bolt loosening in a rotating joint of a custom sewer cleaning vehicle transmission was performed using support vector machines (SVM). Different failures were analyzed for various vehicle operating conditions. Several classifiers were trained to evaluate the influence of the number and location of accelerometers used and to determine the best approach between specific models for each operating condition or a single model for all cases. The results showed that using a single SVM model with data from four accelerometers mounted both upstream and downstream of the bolted joint resulted in more reliable fault detection, with an overall accuracy of 92.4%.

## 1. Introduction

Machine maintenance is a key aspect of ensuring the efficient and safe operation of industrial equipment, preventing sudden breakdowns, and reducing downtime. The maintenance strategy adopted has a major impact on a company’s costs; in fact, if not properly designed, up to one-third of the total cost of maintenance expenditure can be attributed to unnecessary or excessively delayed interventions [1,2,3]. To address these issues, modern maintenance approaches are based on continuous monitoring of machine conditions to detect and respond to failure in a timely manner [4,5,6,7,8]. Based on condition monitoring, three different approaches can be used to detect a failure: model-based, statistical-based, and artificial intelligence-based [9]. Machine learning techniques have emerged as a powerful tool due to their accuracy, robustness, and high computational speed, often leading to superior results compared to the other two condition-based approaches [3,5,10,11]. Furthermore, there are industrial realities where the level of customization of equipment and machines makes statistical and laborious model-based approaches inapplicable, thus requiring the development of machine-learning approaches tailored to the specific case study. This is the case, for example, of customized vehicles, where small batches of vehicles are produced by adding new elements to obtain required functionalities. However, this situation can be extended beyond the automotive sector to all areas where the degree of customization of machines is a predominant factor.

In this context, due to their flexibility and ease of design, bolted joints are broadly used in a variety of industrial applications to connect different structural components. However, these bolted joints can be subjected to a wide variety of load combinations during normal operation and therefore tend to loosen, compromising the structural integrity or full functionality of the particular machine. Furthermore, such problems can be exacerbated when rotating bolted joints are considered, such as those found in heavy rotating machinery and vehicles [12]. For these reasons, the detection of bolt loosening plays an important role in maintenance, especially when it can lead to failure and subsequent machine downtime [13].

There are several techniques that can be used to monitor the condition of bolts: vision-based [14,15,16], percussion-based [17,18,19], electro-mechanical impedance-based or via piezoelectric transducers [20,21,22,23], ultrasonic-based [24,25], and vibration-based [26,27]. Huynh et al. used vision-based techniques by exploiting a deep learning algorithm to understand whether hex head bolts were loose by estimating the angle of rotation with respect to the sound condition [14]. Additionally, using vision-based techniques, Ramana et al. identified the failure based on the exposed shank length of the bolt and then trained a support vector machine (SVM) algorithm for classification [15]. Zhang et al. implemented automated bolt loosening detection based on the exposed length of the threaded bolt using a region-based convolutional neural network to avoid the need to manually extract features from machine vision images [16]. However, these methods require the installation of a camera with a good viewing angle on the bolted connections, which can be difficult in terms of available space and during the actual operating conditions of machines with moving or rotating elements, such as vehicles. Percussion-based loosening detection consists of tapping the bolted joint, recording its acoustic response, processing these signals and extracting characteristic features, such as power spectrum density, and classifying faults using machine learning [17,18,19]. Although suitable for civil structures, this approach is not well suited to continuous monitoring of machines under operating conditions. The other techniques mentioned above for detecting bolt loosening are based on the use of sensors in direct contact with the machine being monitored and are capable of detecting even the early stages of failure, but differ in the principle of measurement.

Fault detection through continuous health monitoring based on vibration measurement and machine learning has been successfully applied to various mechanical elements such as gears and gearboxes [28,29,30,31], bearings [32,33], induction motors [34,35,36], and centrifugal pumps [37]. However, research using this approach for bolt loosening detection is limited [13]. Eraliev et al. used a variety of machine learning classifiers to detect the early stages of loosening in a multi-bolted AC motor by acquiring vibration signals. In this work, the authors employed a short-time Fourier transform to extract features from the raw signals and then performed feature reduction on the obtained spectrograms to improve classification accuracy [27]. Results presented in [27] clearly show that major improvements of the SVM in this context are necessary to achieve sufficient accuracy. Furthermore, most sensor-based studies have focused on civil structures [38,39] rather than machines with rotating bolted joints and have tended to use approaches based on electro-mechanical impedance changes. For civil structures, kernel-based machine learning techniques such as SVM have been successfully applied to damage detection from vibration data by Santos et al. [40]. In addition, most research papers on bolt loosening have developed methodologies using controlled laboratory experiments rather than industrial case studies in a real world environment [6].

In this research work, SVM classifiers were developed to detect the loosening of bolts in a rotating joint of the transmission unit of a sewer cleaning vehicle. Joint vibration monitoring was used to identify two different degrees of joint loosening for different vehicle operating conditions. The influence of the number and the location of the installed accelerometers was investigated, as well as the best approach between specific SVM models or a single model for all operating conditions, finally achieving high classification accuracies.

## 2. Materials and Methods

**Figure 1:**
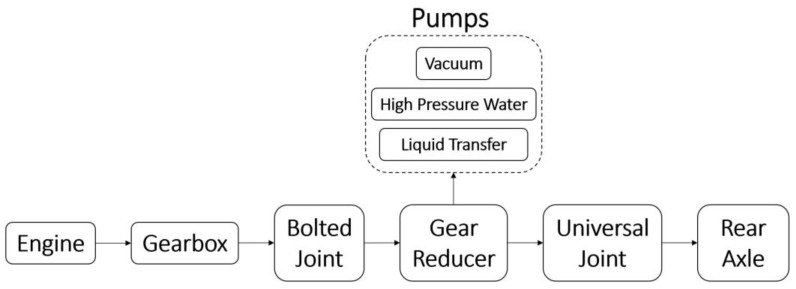
Schematic diagram of the transmission unit of the sewer cleaning vehicle.

A schematic diagram of the transmission unit of the sewer cleaning vehicle under investigation is shown in Figure 1. The rotating joint to be examined consists of four bolts that connect the shaft at the output of the gearbox to the shaft at the input of the gear reducer, which supplies both the rear axle and the pumps. The main gearbox at the engine output consists of six gears. The vacuum, high-pressure water, and liquid transfer pumps are operated by engaging the fourth, fifth, and sixth gears at 720 rpm. These are the operating conditions under which it is necessary to detect the looseness of the bolted connection to avoid failures while the pumps are in use.

### 2.1. Experimental Setup and Data Acquisition

**Figure 2:**
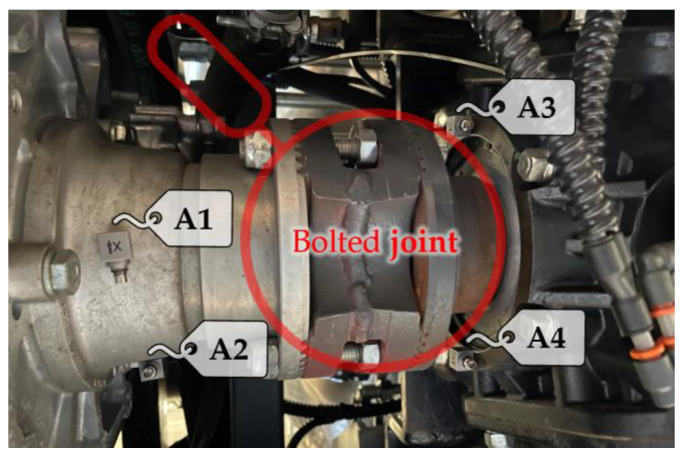
The experimental setup is schematically represented, with four designated measurement locations. Two are located upstream, while the other two are located downstream of the bolted joint under examination.

Figure 2 displays the experimental setup employed to fully capture vibrations related to periodic events caused by shaft rotations and gear angular velocity fluctuations under the selected operational conditions. Our assessment of the system’s response, measuring acceleration, focuses on two locations: one upstream and one downstream of the bolted joint. Two accelerometers (A1 and A2) are placed between the gearbox and the joint, while two others (A3 and A4) are placed between the joint and the gear reducer. The setup incorporates (i) four B&K 4535-B triaxial accelerometers and (ii) an LMS SCADAS Recorder 09 mobile PC-based multichannel analyzer platform, running the LMS Test.Lab 14A suite. This facilitates the acquisition and recording of time histories of the measured accelerations alongside the engine speed. All signals are simultaneously recorded in the time domain with a sampling frequency of 4096 Hz.

In order to detect the appearance of fault conditions, a supervised approach was adopted. Therefore, nine different cases of vehicle operation were established by varying the gears engaged and the type of failure, as reported in Table 1. Two different types of rotating joint failure were established, first by loosening two opposing bolts, condition F1, and then by loosening all four bolts, condition F2. Condition corresponding to normal operation is labeled by G.

### 2.2. Feature Selection

Extracting features from raw vibration signals before feeding them into machine learning models has a significant impact on the performance of the model itself. This step can only be omitted if deep learning techniques are used, but these have a much higher computational cost. Lei et al. found that combining features from both the time and frequency domains was a simple and effective way to detect and identify a fault in a planetary gearbox [31]. In addition, Li et al. developed a deep learning model for fault detection in a gearbox which, by taking the same signal in both the time, frequency, and time-frequency domains as input, achieved higher classification accuracies than models using only one of the domains [30].

In this study, after a settling period, 11 different signals (batches) lasting 10 s each were recorded for every experimental case in a series of different tests. For each of these batches, features were extracted in both the time and frequency domains. Taking both domains into account increases the information power of the signal [30,31,41,42]. In the time domain, the maximum acceleration and the root mean square (*RMS*) were used as features, while moving to the frequency domain using the fast Fourier transform, the amplitude of the maximum peak was considered. Let x(t) be the acquired signal. The features considered can be defined as:

(1) $$Max\;Acceleration=max[x\left(t\right)],$$

(2) $$RMS=\sqrt{E\left[x(t)\right]},$$

(3) Peak=max(|X(ω)|),

where $E\left[x(t)\right]$ is the mean value of the signal, and X(ω) is the Fourier transform of the signal. These features were selected because a deviation from the proper operating conditions of the bolted joint due to loosened bolts results in attenuation of the maximum signal amplitude, a change in the signal dispersion which influences the recorded amplitude of the maximum peak in the frequency domain, and the introduction of other harmonic components which affect the RMS value.

### 2.3. Support Vector Machines Models

The SVM, introduced by Cortes and Vapnik, is a supervised machine-learning model that can classify two different classes of data [43]. This algorithm uses a hyperplane that separates these two different classes of data, thus establishing a decision boundary. The SVM finds the optimal hyperplane by maximizing the margin between the two classes, where the margin refers to the distance between the hyperplane itself and the nearest data point of each class. The data points that lie on the margin, i.e., closest to the hyperplane, are called ‘support vectors’. The hyperplane is defined as:

(4) ω·x+b=0,

where x is the input vector of data points $x_{i}$ (i=1,2,…,N), $\omega \in R^{N}$ is the normal vector to the hyperplane and b∈R. The width of the margin to be maximized is:

(5) $$margin\;width=\frac{2}{\left\|\omega \right\|}.$$

For mathematical convenience, the optimal hyperplane can be obtained by minimizing:

(6) $$\frac{1}{2}{\left\|\omega \right\|}^{2}.$$

When the data points are non-linearly separable, an approach called soft-margin SVM is used to allow for the misclassification of some observations $x_{i}$ which fall in the wrong part of the margin. A slack variable $\xi_{i}(i=1,2,\ldots ,N)$ is introduced into the minimization problem together with a penalty parameter C, which defines a trade-off between the complexity of the hypothesis space and the number of errors allowed:

(7) $$\frac{1}{2}{\left\|\omega \right\|}^{2}+C\sum_{i=1}^{N}\xi_{i}.$$

In addition to the soft-margin SVM, in order to correctly classify non-linearly separable data points while avoiding overfitting, a kernel trick can be used. It exploits Cover’s Theorem, which states that mapping a non-linearly separable input space to a higher-dimensional space makes the linear separation more likely [44]. After using this kernel trick, the optimal hyperplane is defined within this new space, where classification is easier to perform. In this work, the radial basis function (RBF) kernel was used:

(8) $$K\left(x_{j},x_{k}\right)=\exp\left(-\frac{{\left(x_{j}-x_{k}\right)}^{2}}{\sigma^{2}}\right),$$

where $x_{j}$ and $x_{k}$ are two elements of the input vector x and the kernel parameter σ∈R.

SVM can be extended to perform multi-class classifications using mainly two coding designs: ‘one vs. one’ (OVO) and ‘one vs. all’ (OVA) [45,46]. If m is the number of classes, in the OVO approach, m(m−1)/2 binary classifiers are constructed, each trained with data from two of the m classes, and the rest are ignored. Whereas in the OVA approach, m binary classifiers are constructed, each trained with one class defined as positive and the rest of the classes defined as negative. In this research, error-correcting output codes and a loss-weighted decoding scheme were used to aggregate the results of the constructed binary classifiers and generate the predictions [47,48].

Therefore, in a multi-class SVM classifier defined so far, the hyperparameters to be optimized are the penalty parameter C, the kernel parameter σ, and the multiclassification coding design OVO or OVA.

To build the SVM model, the input data set was first shuffled to avoid model conditioning and increase its generalization ability and then divided into a training set and a test set (80–20%). The training set was subsequently normalized using the *z-score*. Let ${\bar{x}}_{TR}$ be the average of the input training data vector $x_{TR}$ and S be the standard deviation, the *z-score* $z_{i}$ of an observation $x_{i}$ $\left(i=1,2,\ldots ,N_{TR}\right)$ is defined as:

(9) $$z_{i}=\frac{x_{i}-{\bar{x}}_{TR}}{S}.$$

Since a real industrial case study is analyzed in this research, it is important to use this normalization method, which not only returns a data set with a mean of 0 and a standard deviation of 1 but is also less affected by the presence of outliers and also preserves the shape properties of the original data set, such as skewness and kurtosis. Afterward, the test set was normalized using the normalization parameters of the training set. In this way, the model is not biased by the data of the test set, which remain unseen until the moment when the generalization ability of the model is evaluated.

To optimize the hyperparameters, avoid overfitting and thus increase the generalization power of the model, a k-fold cross-validation approach with 10 folds was used on the training set [49]. With this approach, the training set is randomly divided into k subsets of equal size. Of these k folds, k-1 is used to train the model, and in the last, called validation, the trained model is evaluated. This cross-validation process is repeated k times, with each of the k subsets used exactly once as the validation set. Finally, the k-validation results obtained are averaged to produce a single estimate. Along with cross-validation, the hyperparameters were optimized using Bayesian optimization and grid search. Bayesian optimization iteratively develops a global statistical model related to the objective function to be minimized. This approach typically uses a Gaussian process model of the objective function, computing a posterior distribution at each iteration using previous evaluations of the objective function and exploiting nonlinear Bayesian regression. An acquisition function is then used to determine the next point in the hyperparameter space where it is most convenient to evaluate the objective function. This is done by finding the point that maximizes the acquisition function. For the sake of brevity, the reader is referred to the reference literature for further details on these optimization methods [50,51]. Bayesian optimization with 30 iterations and “expected improvement plus” as the acquisition function was employed. Subsequently, a 10 × 10 grid search around the found point was used to fine-tune the hyperparameters.

Finally, the unseen test set was used to evaluate the performance of the trained SVM classifier. The confusion matrix, overall accuracy, precision, recall, and F-measure were the metrics used to evaluate the model predictions.

In this research, several SVM models were developed to identify the best approach for detecting bolt loosening. The influence of the number of accelerometers used was evaluated. In addition, the number of gears considered in each model was varied to assess whether a gear-specific approach, with a different classifier for each gear, or a global approach, where a single classifier can make accurate predictions for each gear indiscriminately, would be more suitable. Table 2 shows the 12 models that were constructed, using at least two accelerometers to have sufficient data to be statistically reliable.

## 3. Results and Discussion

**Figure 3:**
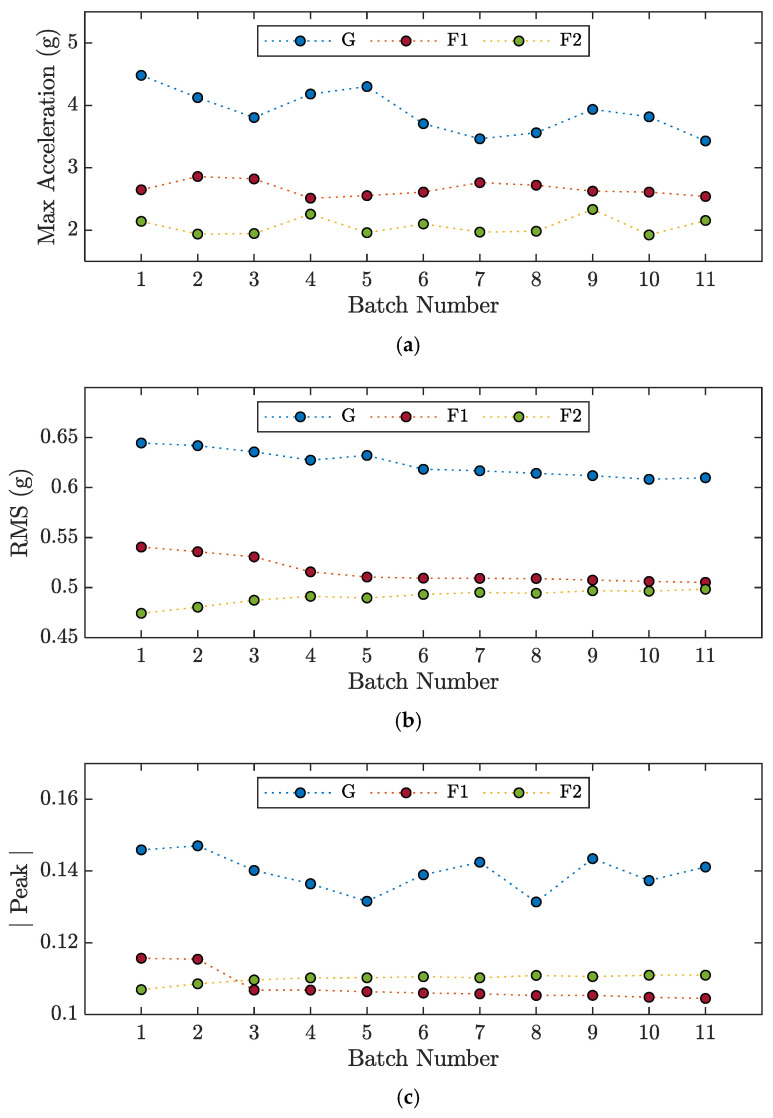
Features evaluated for each batch number of the signals recorded by accelerometer A1 along the *x*-axis during the experiments M4-G, M4-F1, and M4-F2: (**a**) Max Acceleration; (**b**) Root Mean Square; (**c**) Amplitude of the Peak in the frequency domain.

Extracting features from raw signals is useful for algorithms such as SVM because it increases the separability of the different classes to be identified. Figure 3 shows the maximum acceleration, root mean square, and peak amplitude in the frequency domain calculated for different batches of signals acquired from the *x*-axis of accelerometer A1 during experiments M4-G, M4-F1, and M4-F2. From Figure 3, it can be seen how well these three features separate the three different classes. However, the separations are not particularly well defined, especially between classes F1 and F2. The same degree of one-dimensional separation between the classes can also be observed for the other experimental tests and with the other accelerometers, only one of which has been reported so as not to burden the reading.

**Figure 4:**
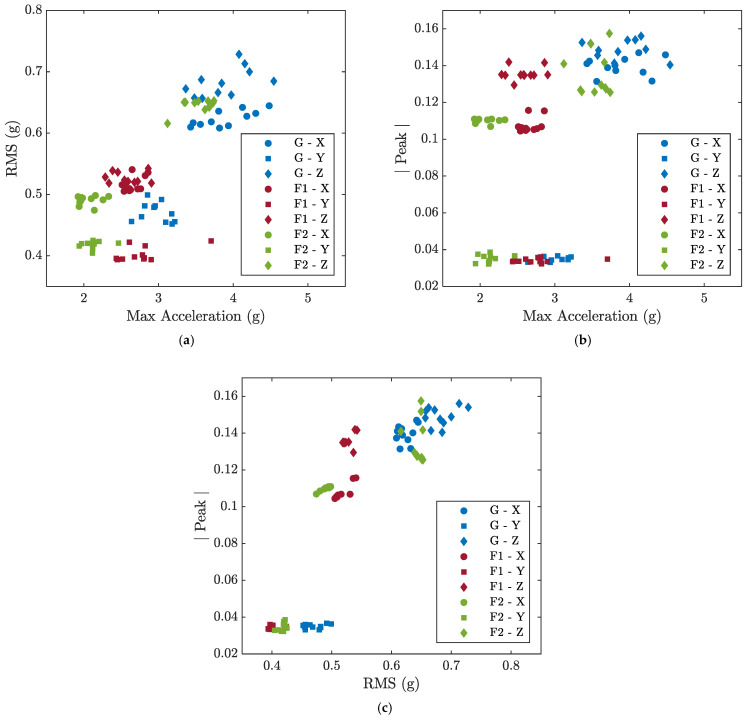
Two-dimensional scatter plots of the extracted features associated with the signals acquired from the X, Y, and Z axes of the A1 accelerometer during the M4-G, M4-F1, and M4-F2 experiments; (**a**) Max Acceleration-RMS; (**b**) Max Acceleration-Peak; (**c**) RMS-Peak.

Figure 4 illustrates the feature space of the signals acquired from the X, Y, and Z axes of the A1 accelerometer during the M4-G, M4-F1, and M4-F2 experiments using two-dimensional scatter plots. From Figure 4, it is clear that the use of these features creates defined clusters of the different classes, but these clusters cannot be separated linearly, necessitating the use of a kernel such as RBF. Furthermore, considering the points relative to the *z*-axis of class F2 in Figure 4b,c, it can be seen that the soft-margin SVM approach is necessary to correctly handle misclassifications.

**Figure 5:**
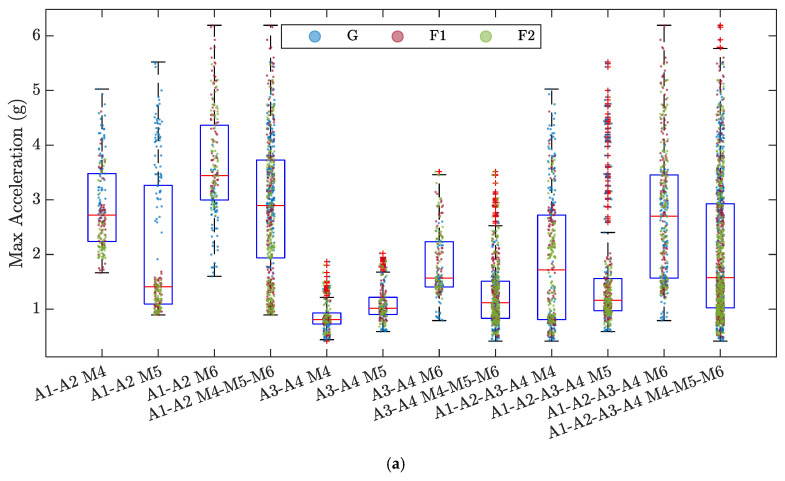

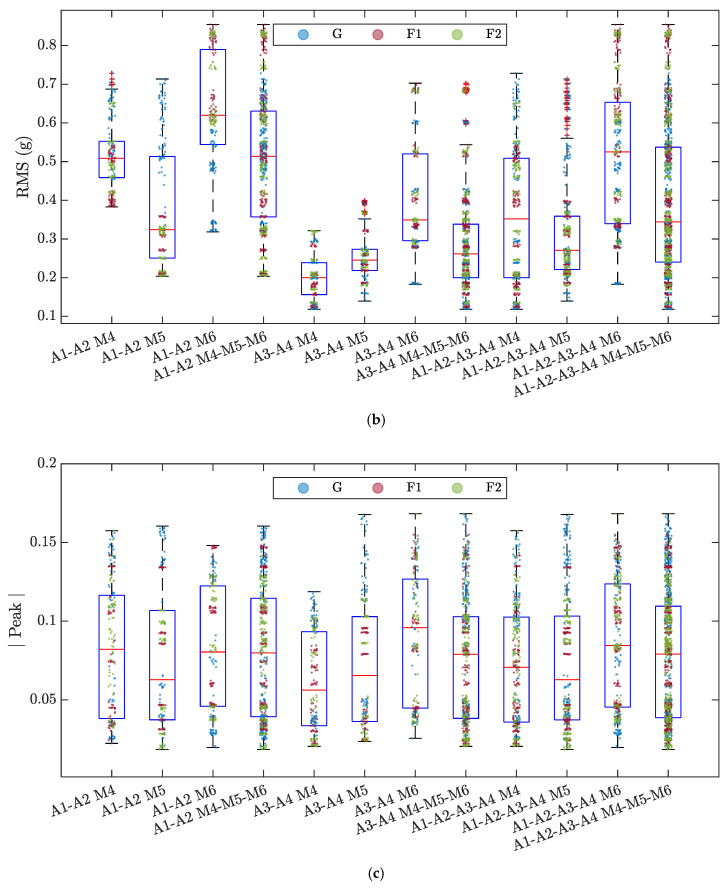
Boxplots of the data distribution of each feature for the 12 SVM models developed: (**a**) Max Acceleration; (**b**) RMS; (**c**) Amplitude of the Peak in the frequency domain. Red horizontal lines show the median of each distribution. From the box, whiskers extend to the most extreme observation within 1.5 times the interquartile range (equal to the distance between the 75th and 25th percentiles). Outliers are indicated by a red plus symbol. Within each boxplot, the corresponding data are illustrated and colored according to the three classes G, F1, and F2.

The data distributions of each feature are described using boxplots for all the 12 SVM models developed (Figure 5). The interquartile range (IQR), the difference between the 75th and 25th percentiles, of the maximum acceleration and RMS distributions varies significantly as the number and position of the accelerometers considered and the gear engaged vary. In particular, when considering the data obtained from accelerometers A3 and A4, located downstream of the bolted joint under consideration, this descriptor is significantly reduced compared to the other cases, indicating a lower spread of the data. Furthermore, the models built using only the data from these two accelerometers have the highest number of outliers, which can negatively affect the model performance. In contrast, for the peak amplitude feature, less variability in the IQR is observed between the data sets of the different models. To enrich the analysis of the data distributions for each of the constructed SVM models, the data have been added to the boxplots and colored according to the three classes G, F1, and F2. In this way, it is possible to see that the RMS and the peak amplitude present distributions formed by many clusters of the different classes, while for the maximum acceleration, the distributions are continuous with less clear separations between the different conditions of joint loosening.

Table 3 reports the overall classification accuracy, precision, recall, and F-measure of each SVM model built, along with the numerosity of the test set on which the performance evaluation was carried out. The models trained with data from the two accelerometers downstream of the rotating bolted joint (A3-A4) are the worst-performing models, with accuracies even below 90 percent. In fact, performance is lowest with either a gear-specific approach or a global approach that considers all gears together. By contrast, using data from the accelerometers upstream of the bolted joint, the gear-specific approach has high accuracies of 94.9%, 100.0%, and 94.9% for the fourth, fifth, and sixth gears, respectively. Nevertheless, the accuracy of the model using accelerometers A1 and A2 drops to 90.7% when the data from all three engaged gears are used as input. On the other hand, exploiting data from all four accelerometers, the gear-specific models achieve high accuracies of 87.3%, 91.1%, and 93.7% for the three engaged gears, and the global approach also proves to be high-performing with an accuracy of 92.4%. In addition, all models exhibit balanced precision and recall scores, with F-measure performance equal to or better than overall accuracy.

However, it is also important to note the sample size on which the performance of the constructed models was evaluated. Although the gear-specific approach using the two accelerometers upstream of the bolted joint seems to give the best results, the small test set on which it was evaluated should be mentioned. In general, SVMs maintain high classification accuracy when the data sets involved are small, up to a few thousand observations at most [7]. Nonetheless, within these limits, the availability of a larger amount of data leads to a better generalization ability of the model. In this context, the model that uses all accelerometers together and is able to detect defects for all three gears indiscriminately proves to be more reliable, even if the overall accuracy is slightly lower. Furthermore, in order to understand which solution is best suited to the case study under consideration, it is possible to compare this model with gear-specific models (with A1-A2) by exploiting other metrics such as confusion matrix, accuracy, and recall.

**Figure 6:**
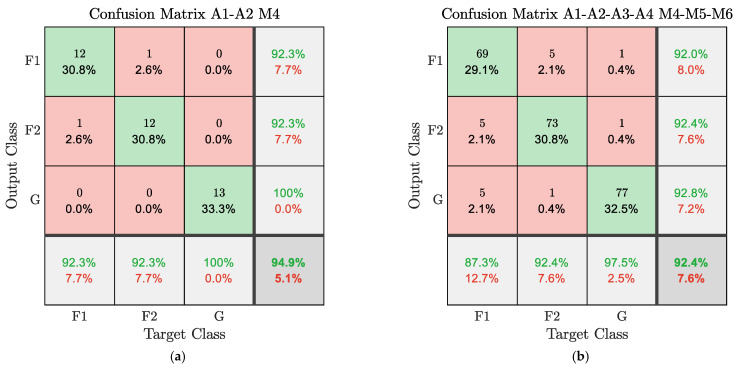
(**a**) Confusion matrix of model A1-A2 M4; (**b**) Confusion matrix of model A1-A2-A3-A4 M4-M5-M6; for each class, the precision scores are given in the rightmost column, the recall scores in the bottom row, while the overall accuracy is given in the bottom right box.

Figure 6 shows the confusion matrices, together with the accuracy and recall scores for each class and the overall accuracy of one of the gear-specific models, the A1-A2 M4, and the model with the more general approach, A1-A2-A3-A4 M4-M5-M6. It can be seen that the results between the two models are comparable, especially for the precision score, which is related to the occurrence of false positives, which need to be avoided or minimized in the industrial case under investigation.

From the performance shown in Table 3, the SVM algorithm has proven reliable for detecting bolt loosening from vibration signals. Compared to the proposed approach, in [27], the short-time Fourier transform (STFT) was used to extract features from the raw signals. Each frequency was considered a feature, and the change in the STFT over time formed the dataset. Multiple classification algorithms were tested, but the accuracy of the SVM was only about 60%. This difference in classification performance could be attributed to two main factors: the selected features and the size of the dataset. In [27], the SVM algorithm was trained on approximately twenty thousand data points. Such a dataset size is likely too large for the SVM classifier to handle, resulting in a high training time and low performance. In addition, in [27], only frequency-based features were used, while in the proposed approach, both time and frequency domain features are considered, which helps to increase the performance of the algorithm. In [21], multi-bolt joint loosening detection was performed by using piezoelectric transducers through the active sensing method instead of using vibration signals. Least square SVM was applied to exploit a damage index based on multivariate multi-scale fuzzy entropy, and classification accuracies of 89% and 96% were achieved on two different multi-bolt connections. This corroborates the results obtained with the proposed global approach, which exploits a different physical principle and the same machine learning algorithm, and yields a comparable overall accuracy of 92.4%.

**Figure 7:**
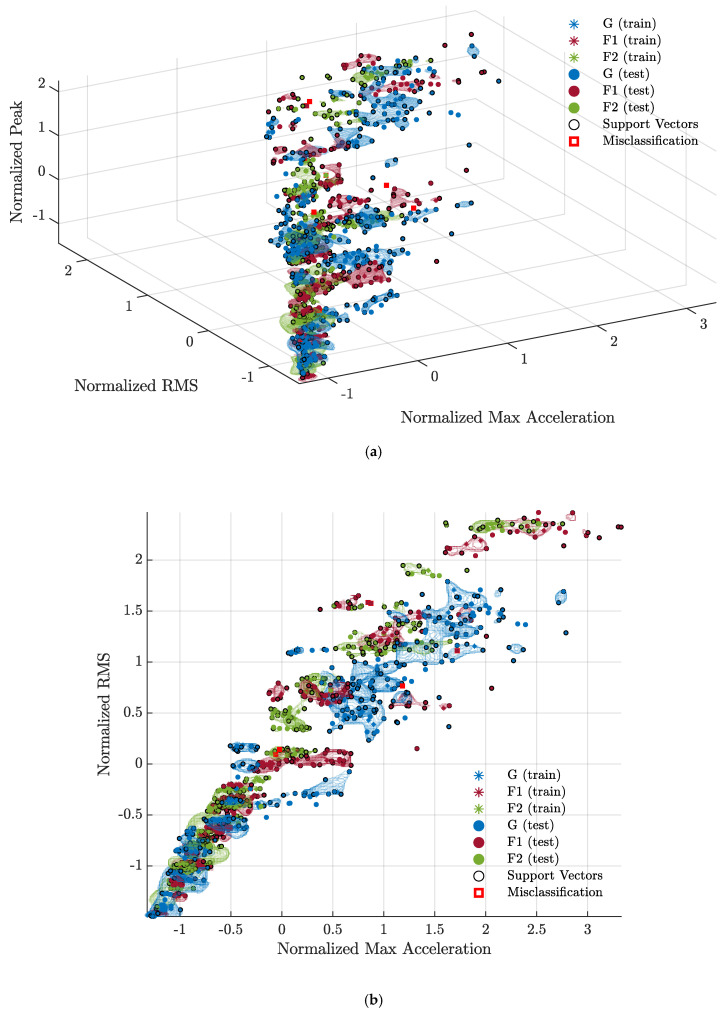

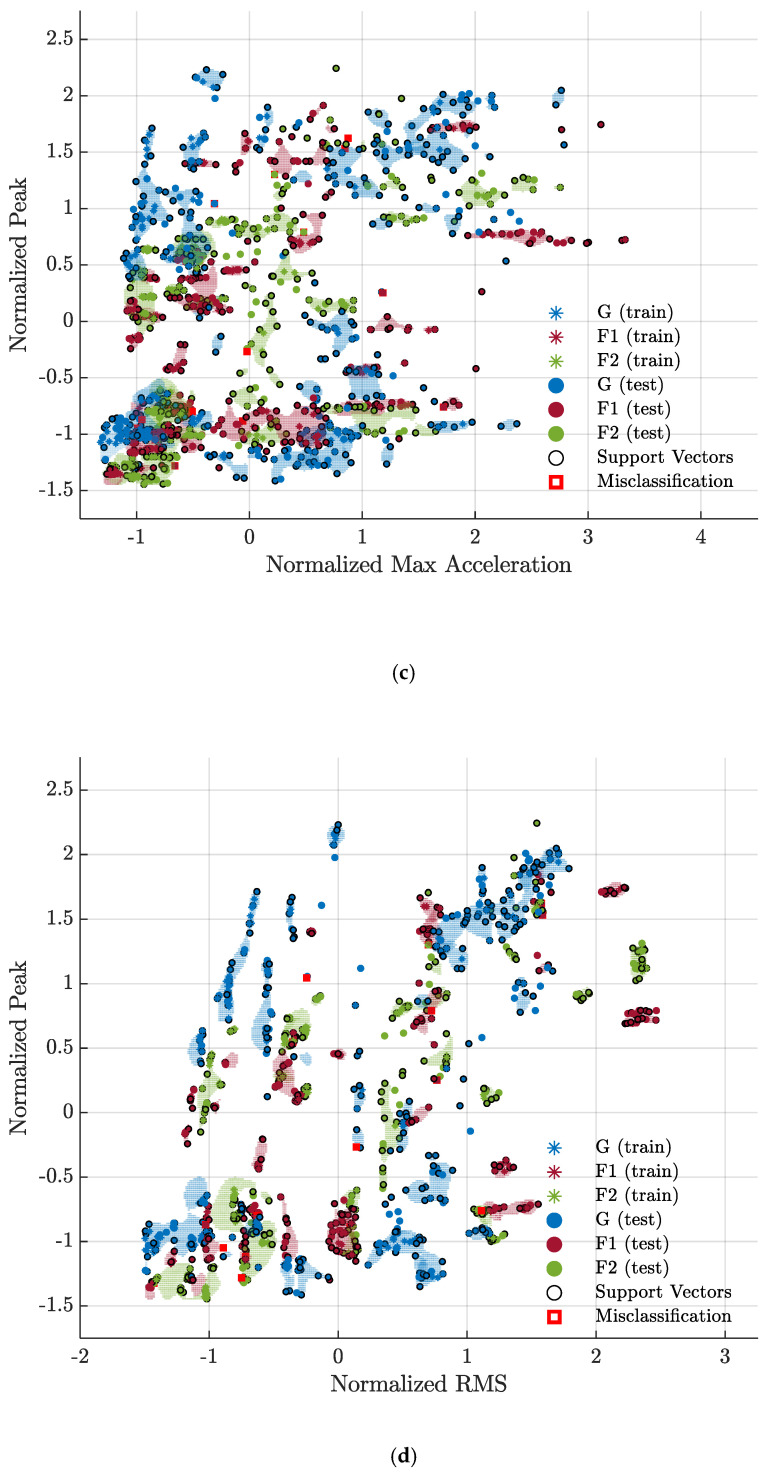
Scatter plots of the predictions of the trained A1−A2−A3−A4 M4−M5−M6 SVM model: (**a**) three-dimensional view of the feature space; (**b**) Normalized Max Acceleration—Normalized RMS; (**c**) Normalized Max Acceleration—Normalized Peak; (**d**) Normalized RMS—Normalized Peak. Training data are indicated by stars, while test data are indicated by solid dots. Among the training data, the support vectors are surrounded by a circle. Incorrectly classified data are framed by a red square. The regions of the data space enclosed by the support vectors, in which the predictions of the multi-class model have the highest probability using the loss-weighted decoding scheme, are colored according to the corresponding class [47,48].

Finally, Figure 7 illustrates the normalized data space of the SVM model A1-A2-A3-A4 M4-M5-M6, showing training and test data, support vectors, and misclassified observations (framed by a red square). It can be seen that the machine learning approach used in this research work is particularly effective in defining the non-linearly separable regions of the space associated with each class (indicated by the colored lines) bounded by the support vectors.

## 4. Conclusions

In this paper, an SVM classifier has been developed to detect different failure conditions due to the loosening of bolts in a rotating joint in the transmission unit of a sewer cleaning vehicle. Condition monitoring of the bolted joint was performed using vibration measurements under two different failure scenarios (two or four loose bolts) and under healthy operating conditions. Four triaxial accelerometers were installed, two upstream and two downstream of the joint. Three types of vehicle working conditions corresponding to the engagement of different gears were analyzed.

Both time and frequency domain features were extracted from the raw signals to enhance the performance of the SVM classifier. Several models were trained to evaluate the influence of the number and location of the accelerometers used and to determine the best approach to fault detection between gear-specific models with a classifier for each gear or a more general model capable of making predictions for all gears engaged. All models developed were trained using a 10-fold cross-validation approach to avoid over-fitting and to increase the generalization power of the models themselves, also given the small size of the datasets. The results showed that an approach using data from all four accelerometers, mounted both upstream and downstream of the bolted joint, to detect failures for all different vehicle operating conditions produced the most reliable predictions, with an overall accuracy of 92.4% and, in particular, high precision, with values greater than 92% for each class.

The machine learning approach developed in this research has proven to be able to identify different types of failures in a real industrial environment, using a methodology suitable for dealing with small datasets typical of small to medium industrial realities while maintaining excellent generalization capabilities in unseen cases. However, for larger datasets, other classification algorithms may be more appropriate than SVM. Several future directions of research can be identified. First of all, an extension of this approach could introduce more complex signal descriptors, such as Renyi’s entropy [52] and the Lempel–Ziv complexity [53], to evaluate their possible influence in improving classification accuracy. As the number of features considered increases due to the black-box nature of SVM classifiers, a further development would be to perform feature ranking to identify those features that have the most significant impact on the performance of the SVM algorithm. Moreover, the output from the classifier could be used to design a predictive maintenance protocol with the scope to optimize maintenance operations. Finally, the influence of the size of the dataset given as input to the classifier could also be evaluated by acquiring more experimental data. In particular, the performance of the SVM classifier could be compared with the performance of other algorithms, such as neural networks and random forests, which could capture more complex patterns in larger datasets.

## Figures and Tables

**Table 1:** Experimental cases for fault detection.

Case ID	Engaged Gear	Fault Description	Class
M4-G	4th	No fault	G
M5-G	5th	No fault	G
M6-G	6th	No fault	G
M4-F1	4th	Two opposite bolts are loosened	F1
M5-F1	5th	Two opposite bolts are loosened	F1
M6-F1	6th	Two opposite bolts are loosened	F1
M4-F2	4th	All four bolts are loosened	F2
M5-F2	5th	All four bolts are loosened	F2
M6-F2	6th	All four bolts are loosened	F2

**Table 2:** SVM models developed.

SVM Model	Gears	Accelerometers	Dataset Size
A1-A2 M4	4th	A1 and A2	198
A1-A2 M5	5th	A1 and A2	198
A1-A2 M6	6th	A1 and A2	198
A1-A2 M4-M5-M6	4th-5th-6th	A1 and A2	594
A3-A4 M4	4th	A3 and A4	198
A3-A4 M5	5th	A3 and A4	198
A3-A4 M6	6th	A3 and A4	198
A3-A4 M4-M5-M6	4th-5th-6th	A3 and A4	594
A1-A2-A3-A4 M4	4th	A1, A2, A3 and A4	396
A1-A2-A3-A4 M5	5th	A1, A2, A3 and A4	396
A1-A2-A3-A4 M6	6th	A1, A2, A3 and A4	396
A1-A2-A3-A4 M4-M5-M6	4th-5th-6th	A1, A2, A3 and A4	1188

**Table 3:** Classification accuracy, precision, recall, and F-measure of the SVM models developed.

SVM Model	Test Set Size	Overall Accuracy	Overall Precision	Overall Recall	Overall F-Measure
A1-A2 M4	39	94.9%	94.9%	94.9%	94.9%
A1-A2 M5	39	100.0%	100.0%	100.0%	100.0%
A1-A2 M6	39	94.9%	95.0%	94.9%	95.0%
A1-A2 M4-M5-M6	119	90.7%	90.7%	90.7%	90.7%
A3-A4 M4	39	92.3%	93.8%	92.3%	93.0%
A3-A4 M5	39	79.5%	84.6%	79.5%	82.0%
A3-A4 M6	39	82.1%	82.5%	82.0%	82.3%
A3-A4 M4-M5-M6	119	89.8%	90.0%	89.9%	90.0%
A1-A2-A3-A4 M4	79	87.3%	88.0%	87.5%	87.7%
A1-A2-A3-A4 M5	79	91.1%	91.2%	91.2%	91.2%
A1-A2-A3-A4 M6	79	93.7%	93.8%	93.7%	93.7%
A1-A2-A3-A4 M4-M5-M6	237	92.4%	92.4%	92.4%	92.4%

## Data Availability

Not applicable.
